# 206. A Cluster Randomized Trial Evaluating Universal Gloving for Reducing *Clostridioides difficile* Acquisition and Infection in VA: The GloRI Study

**DOI:** 10.1093/ofid/ofae631.064

**Published:** 2025-01-29

**Authors:** Linda McKinley, Cara E Ray, Julie Keating, Noreen Brennan, Christopher J Crnich, Dembry Louise, Jonah Dixon, Charlesnika T Evans, Michael Gelman, Daniel J Morgan, Helene Moriarty, Florine Ndakuya-Fitzgerald, Susan M Pacheco, Christopher D Pfeiffer, Amy Weintrob, Nasia Safdar

**Affiliations:** Wm. S. Middleton Memorial VA Hospital, Madison, Wisconsin; Edward Hines, Jr. VA Medical Center, Chicago, Illinois; Madison VA Hospital, Madison, Wisconsin; Bronx VA, Bronx, New York; University of Wisconsin School of Medicine and Public Health, Madison, WI; West Haven VA, West Haven, Connecticut; University of Wisconsin - Madison, Madison, Wisconsin; Northwestern University and VA, Hines, Illinois; Bronx VA Hospital, Bronx, New York; University of Maryland School of Medicine, Baltimore, MD; Villanova University, Philadelphia, Pennsylvania; Milwaukee VA Hospital, Milwaukee, Wisconsin; Edward Hines, Jr. VA Hospital and Loyola University Medical Center, Hines, Illinois; VA Portland Health Care System, Portland, Oregon; Washington DC VA Medical Center/ George Washington University, Washington, District of Columbia; University of Wisconsin School of Medicine and Public Health, Madison, WI

## Abstract

**Background:**

*Clostridioides difficile* infection (CDI), the most common healthcare associated infection (HAI) in U.S. hospitals, causes half a million infections and 30,000 deaths annually. Prevention of hospital-onset (HO) CDI has quickly become a priority for most hospitals. Hospital exposure to *C. difficile* can occur through cross-contamination of healthcare personnel (HCP) hands or through shared environmental surfaces or equipment. Infection prevention measures focus on HCP hand hygiene and barrier precautions (i.e., gowns and gloves) and cleaning of environmental surfaces, especially for patients with known CDI. However, colonized patients can also serve as a reservoir for cross-contamination, and microbiological screening for *C. difficile* colonization is not routinely performed in healthcare.

**Figure:**
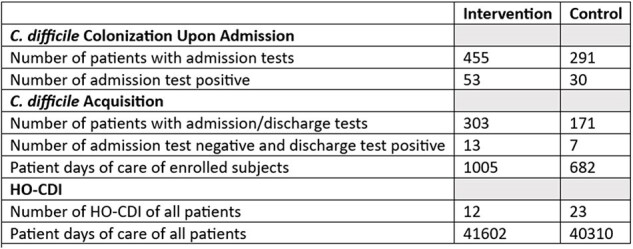

**Methods:**

We conducted a cluster randomized trial (CRT) in 10 inpatient units at 5 VA hospitals between April 2022 and December 2023 to evaluate the effectiveness of healthcare worker use of gloves for *all* patient contact (i.e., universal gloving) in reducing acquisition of *C. difficile* colonization (defined as negative PCR at admission and positive PCR at discharge) and HO-CDI (per NHSN LabID criteria, as adjudicated by facility infection prevention personnel for routine quality reporting).

**Results:**

Of 9779 admissions, 780 consented to specimen collection. Table 1 displays preliminary data. At admission, 11.6% (intervention group) and 10.3% (control group) of patients tested were colonized with *C. difficile*. There were 474 patients with both admission and discharge perirectal swabs cultured for *C. difficile*. The *C. difficile* colonization acquisition rate was 12.9 per 1000 patient days of care (PDOC) for the intervention group and 10.3 for the control group (p=0.79). Unit-level aggregate data for the HO-CDI rate was 0.29 per 1000 PDOC for the intervention group and 0.57 for the control group (p=0.07).

**Conclusion:**

No universal reduction in the acquisition of *C. difficile* was detected in acute care units implementing a gloving intervention compared with standard practice, but only 5% (474/9779) of admissions had complete testing data. A numerically lower but statistically nonsignificant HO-CDI rate was detected in the universal gloving group compared to standard practice.

**Disclosures:**

**Christopher D. Pfeiffer, MD, MHS**, Department of Defense/Medpace: Grant/Research Support|Pfizer: Grant/Research Support

